# Raman spectroscopy on live mouse early embryo while it continues to develop into blastocyst *in vitro*

**DOI:** 10.1038/s41598-019-42958-5

**Published:** 2019-04-29

**Authors:** Elena Perevedentseva, Alexander Krivokharchenko, Artashes V. Karmenyan, Hsin-Hou Chang, Chia-Liang Cheng

**Affiliations:** 1 Department of Physics, National Dong Hwa University, Hualien, Taiwan; 2 N. N. Semenov Institute of the Chemical Physics, Russian Academy of Sciences, Moscow, Russia; 3 Department of Molecular Biology and Human Genetics, Tzu Chi University, Hualien, Taiwan; 4 P. N. Lebedev Physical Institute of the Russian Academy of Sciences, Moscow, Russia

**Keywords:** Analytical biochemistry, Nanoscale biophysics

## Abstract

Laser based spectroscopic methods can be versatile tools in investigating early stage mammalian embryo structure and biochemical processes in live oocytes and embryos. The limiting factor for using the laser methods in embryological studies is the effect of laser irradiation on the ova. The aim of this work is to explore the optimal parameters of the laser exposure in Raman spectroscopic measurements applicable for studying live early embryos *in vitro* without impacting their developmental capability. Raman spectra from different areas of mouse oocytes and 2-cells embryos were measured and analyzed. The laser power and exposure time were varied and further embryo development was evaluated to select optimal conditions of the measurements. This work demonstrates safe laser irradiation parameters can be selected, which allow acquisition of Raman spectra suitable for further analysis without affecting the early mouse embryo development *in vitro* up to morphologically normal blastocyst. The estimation of living embryo state is demonstrated via analysis and comparison of the spectra from fertilized embryo with the spectra from unfertilized oocytes or embryos subjected to UV laser irradiation. These results demonstrate the possibility of investigating preimplantation mammalian embryo development and estimating its state/quality. It will have potential in developing prognosis of mammalian embryos in assisted reproductive technologies.

## Introduction

Investigation of early mammalian development using various methods has an aim to understand fundamental mechanisms underlying the process. From practical point of view, obtained results can give important information for mammalian embryo evaluation and selection in assisted reproductive technologies (ART). Recently, evaluation and analysis of ova quality in mammalians *in vitro* fertilization (IVF) to limit embryo overproduction and to improve the outcomes of oocyte and embryos cryostorage programs are getting increasing attention from embryologists^1^.

A number of methods exist for live early mammalian embryos (EME) quality diagnosis and development prognosis^2^. Usually, morphological observation can be used for direct evaluation of live embryos; recently a new method named morphokinetics or observation of developmental dynamics by embryo time-lapse monitoring^3^ has been proposed and demonstrated. Indirect embryo studies are performed by methods such as metabolomics or metabolite profiling using culture medium which may correlate with biochemical state and therefore with developmental potential of embryos^4,5^. Morphological and metabolomics studies don’t affect the survival capacity of oocytes and embryos. However, these methods don’t allow investigation of cytoplasmic organelles or nuclear components distribution, development and monitoring biochemical processes *in situ*; in addition, they are not suitable for quantitative evaluation. Other methods for more precise and solid analyzing of EME state require fixation or even complete destroying of objects and therefore cannot be used for live ova. Methods which allow study of antigens or protein localization by labeled antibodies specific binding, immunohistochemistry; or of biochemical processes, gene expression and protein profiles, such as in biochemical and molecular biological methods are destructive. Therefore, develop a new, minimum-invasive and informative method of EME estimation is needed and topical. Modern optical-spectroscopic methods could be ideal candidates. Advanced laser techniques, such as Raman spectroscopy and its modifications^6–8^, fluorescence spectroscopy and microscopy^7,9^, fluorescence lifetime imaging microscopy (FLIM)^10,11^, fluorescence resonance energy transfer (FRET)^11^, harmonic generation microscopy^12^, and Fourier Transform Infrared Spectroscopy (FTIR)^13^ allow the study of organelles organization, structural and molecular biochemical processes inside living cells without disturbing the living processes.

Raman spectroscopy is a method based on inelastic scattering of monochromatic light. The observed and analyzed frequency shift of the scattered light is directly related to the structural and molecular properties of the material, providing characteristic of the material “fingerprint”^14,15^. Therefore, Raman spectroscopy can be employed to detect the biochemical changes during the process of development. The limiting factor for the laser-based analytical methods is the adverse effect of laser on the investigated biological sample depending on laser wavelength and dose of laser radiation. While the laser irradiation on biological objects such as cells, tissues, organs are studied recently^15,16^; for embryos, some studies have been performed^17^, but more investigation are still required. The main laser effects on early embryos are described^18^. DNA damage, failed embryo development and possible congenital disorders^19^ have been observed as a result of thermal adverse influence^20^ and chemical effects, like generation of singlet oxygen and oxidative stress^18^. On the other hand, thermal effects can be controllable used in IVF technologies^18^. In this study, we investigate the possibility of the embryos subjected to continuous wave (CW) laser irradiation and preserve the developmental ability into blastocyst. Note, the thermal effect of the low power CW laser used is presumably low^21^.

Raman spectroscopy has been applied in studies in reproductive medicine for analyzing the tissues of reproductive system. The methods using Raman spectroscopy for estimation and monitoring in reproduction  technologies have been considered^17^. Although previous spectroscopic studies such as FTIR^22^ and Raman^23,24^ have been performed on mouse oocytes and early embryos, but these works were limited by their investigation on fixed rather than live ova. The fixation procedure can affect and change the object; moreover, the obtained results depend on fixation methods^25^ (and references therein) and the differences between Raman spectra of aldehyde fixed and non-fixed mouse oocytes are demonstrated^26^. However, the first practical application has shown Raman spectroscopy as a valuable tool for identification biochemical markers of oxidative damage^27^. Methods of analyzing the dynamics of biochemical modifications on the example of cortical F-actin in oocyte and conformational changes in protein and glycoprotein secondary structure of the zona pellucida structure^28,29^ were suggested for estimation of cryopreserved matured ovine oocytes *in vitro*; and the laser parameters used, e.g. laser power up to 50 mW and 25 mW at 532 nm excitation wavelength can definitely affect the live embryos^27,28^.

A variation of Raman imaging based on coherent anti-Stokes Raman scattering (CARS) was used for preliminary demonstration of the possibility to quantify lipid content of oocytes from various species^30,31^. The attempt to avoid damaging by environmental stressors or the imaging process itself in analyzing living oocyte is mentioned^2^, but the mechanism of the damage still remains unstudied. Additionally, optical-spectroscopic studies on living mouse oocytes and embryos redox metabolism was investigated via monitoring functional/conformational states of intracellular nicotinamide adenine dinucleotide (in the form of NADH and NAD(P)H) and thiol tripeptide glutathione by measurements of their autofluorescence^32^. FRET method has been used to examine mitochondrial lipid droplet co-localization in live but stained pig oocytes^33^; however the embryo viability after the study also was not estimated.

Recently Raman spectroscopy was applied in embryo studies as a non-invasive tool and for live mouse oocytes and embryos^26,34^, however the absence of independent analysis of the oocyte or embryo state/quality before and after the Raman investigation doesn’t allow reaching conclusion about the method’s safety. Moreover, ova were frozen-thawed at different stages and may hinder their developmental ability after thawing^34^. Without an analysis of developmental ability and estimation of state of the Raman investigated ova, simple “morphological criteria”, may not reveal exactly what the measured Raman spectra demonstrate. The impact of laser irradiation on the inside of the live cell remains undecided.

Successful use of other optical laser method for study of live mouse oocytes and embryos has also been presented^35^. Combining two different higher-harmonic generation modalities, second harmonic generation (SHG) and third harmonic generation (THG), allowed visualizing the subcellular structures, and moreover, the embryos development *in vitro* after the study. Furthermore, offspring were obtained after transfer of the studied embryos to foster mother. We therefore suppose, using safe conditions of a laser irradiation, laser methods can be useful tools to investigate cytoplasmic components distribution as well as biological processes in live oocytes and embryos. Thus, the main aim of this work is to determine the optimal conditions and parameters of Raman spectroscopy applicable for studying live embryos without impacting their developmental capability. The safe parameters of the laser irradiation should allow acquisition of Raman spectra suitable for further analysis. It is hope that Raman measurements under selected conditions are not affecting the early mouse embryo development *in vitro*. The development was observed up to morphologically normal blastocyst, this stage is crucial in all types of manipulations with mammalian early embryos *in vitro*^36,37^.

## Results

### Embryo viability after Raman spectroscopy

Live two-cell embryos were subjected to Raman measurements at different exposure with 532 nm wavelength laser excitation and then cultured for 72 h; then their *in vitro* development to blastocysts was analyzed. The development was characterized via estimation of the embryo ability to reach morphologically normal blastocyst stage and counting the cell number in the embryo using microscopic morphological and fluorescence analysis. Safety conditions were found which allow embryo development after the Raman measurement. The optimal embryo developmental ability was observed at the Raman measurements with laser power up to 3 mW in the focal spot, exposure time 40–55 sec, number of the spectra measured from one embryo 4–6 times (or, for one cell 2–3). The development at these conditions was comparable to the control. The embryos at the blastocyst stage after Raman investigation and control appeared to be completely normal and displayed no observable morphological differences. The rate of development was calculated as the percentage of blastocysts developed from the total number of laser treated or control embryos. These conditions were both the safest for the embryo and allowed measurements of spectra with acceptable signal/noise ratio. Although the rate of blastocyst development of studied embryos was slightly lower than in control, the differences in surviving were not significant (Table 1).

**Table 1:** *In vitro* development of two-cell embryos after Raman spectroscopy.

Experimental groups	Number of embryos	Blastocyst after 72 hr
Raman spectroscopy	27	85.2% (23/27)
Control	30	93.3% (28/30)

**Figure 1:**
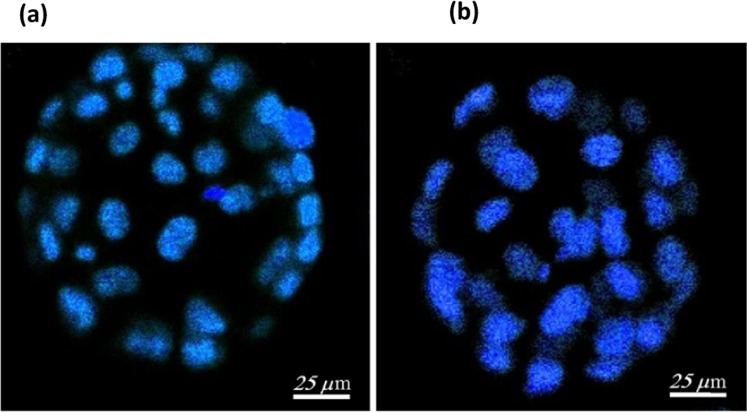
Estimation of the embryo *in vitro* development after Raman investigation. A typical two-photon fluorescence imaging of (**a**) control, (**b**) Raman studied sample. The embryos cell’s nuclei were stained with Hoechst33342 after 72 h *in vitro* cultivation to count the cell under two-photon excitation with 740 nm wavelength femtosecond laser.

The cell numbers in blastocysts developed *in vitro* from untreated and Raman studied two cell embryos were estimated by nuclei counting. Two-photon excitation microscopy was used for the cell counting (stained nuclei of cells). In Fig. 1, the possibility to estimate the cell number using two-photon microscopy is demonstrated. The images of nuclei stained with Hoechst 33342 were recorded with 2-photon excitation using a 740 nm wavelength femtosecond laser. The imaging was used for quantitative count of cells. Although the rate of blastocyst development of laser-irradiated embryos was only slightly lower than in control, the number of cells in blastocysts from the laser treated embryos was observable lower than in the control embryos with P < 0.05 (Table 2).

**Table 2:** Number of cells in blastocysts from Raman studied and control embryos.

Experimental groups	Number of blastocysts	Mean number of cells
Raman studied	19	31.2 + 10
Control	18	46.2 + 19.01

### Raman spectroscopic investigation of live embryos

**Figure 2:**
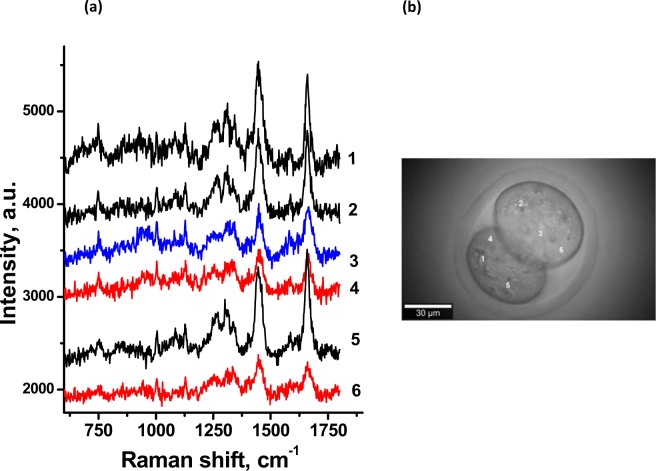
Raman spectroscopic measurements of 2-cells living embryo. (**a**) Raman spectra measured from different areas of the individual embryo: 1, 2, 5 (dark cytoplasmic area); 3 (nucleus); 4, 6 (light cytoplasm). Excitation 532 nm; power in focal spot 3 mW, 60× WI objective; acquisition time of 1 spectrum was 40 sec. (**b**) The image of the studied embryo with marked areas of measurements.

**Figure 3:**
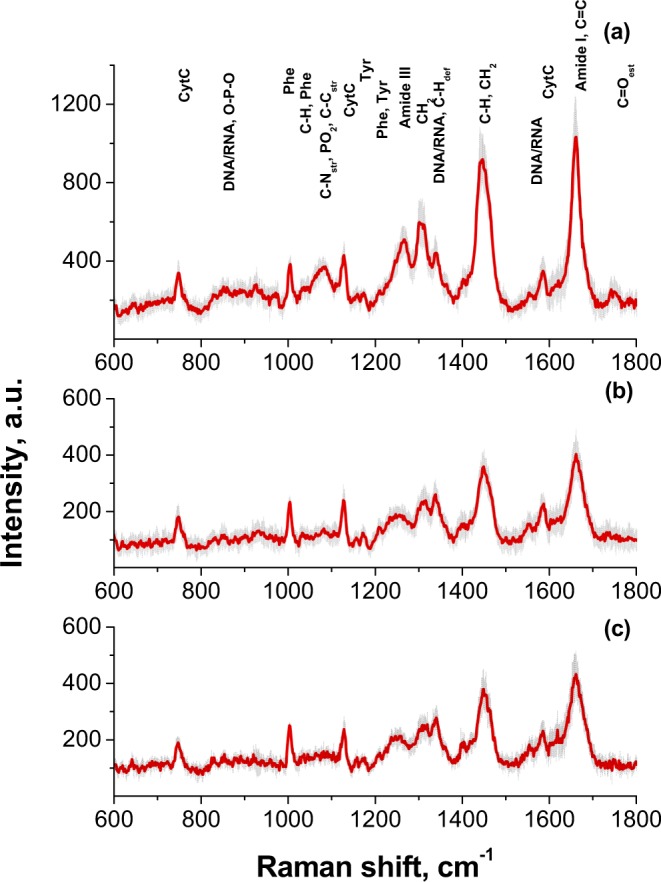
Characteristic averaged Raman spectra (1) measured from (**a**) lipid-rich (dark cytoplasm) area, (**b**) (light) cytoplasm, (**c**) nucleus of the embryo. Excitation laser wavelength is 532 nm; power 3 mW; 60× WI objective. The averaging was based on 25 spectra measured from corresponding areas of 7 embryos. Standard deviations are shown.

Figure 2a shows the characteristic Raman spectrum of the individual embryo; pronounced Raman bands characteristic of most living cells^38,39^ are observed. Image in Fig. 2b demonstrates morphologically good mouse 2-cell stage embryo subjected to measurement. It reveals blastomeres of equal size without cytoplasmic fragments in normal perivitelline space and with regular zona pellucida. The laser was focused on three morphologically distinct areas (1–6 in Fig. 2b), and spectra measured from the same embryo are shown (Fig. 2a): dark cytoplasmic areas (spectra 1, 2, 5), nuclei (spectrum 3), and light cytoplasm (spectra 4, 6). Every selected area has its characteristic spectrum. While the spectra measured in the same morphological areas are very similar not only for one embryo but for different embryos kept in the same conditions, the spectra for different morphological areas are significantly different. For better distinguishing the cell chemical components, characteristic Raman spectra for different areas of the embryo were averaged and compared (Fig. 3); every spectrum is averaged from 8–10 spectra obtained at exactly the same conditions from corresponding morphological areas of 7 (survived) developing embryos. Figures 2 and 3 represent the characteristic Raman bands for living cell; for different investigated areas the bands positions and relative intensities can differ. As seen from the spectra, one of the most pronounced bands is centered near 1445 cm^−1^. It is originated mostly from lipids and associated with the C-H deformation. Near this spectral position, the CH_2_ bending vibration and CH_3_ deformation are also observed, originated both from the protein and lipids. Other pronounced band, also of the protein and lipids, is near 1660 cm^−1^ and is assigned to amide I vibrational mode of peptide bonds in proteins and to C=C stretching in unsaturated lipids and carbohydrates. The band centered near 1258 cm^−1^ is also from protein, the amide III band. Note that the amide I and amide III are sensitive to protein conformation and can be used for the protein conformation analysis subjected to various treatments^40^. The regions predominately associated with lipid bonds vibrations include bands between 1430–1465 cm^−1^, bands between 1020–1140 cm^−1^ resulting mainly from the C-O stretching and C-O-H bending modes of carbohydrates; and bands in the 1290–1310 cm^−1^ range, particularly, the CH_2_ twist, which occurs in lipid molecules at 1301 cm^−1^, the C-H deformation at 1340 cm^−1^. The wide band in the 2800–3000 cm^−1^ range is overlapping of symmetric stretching vibrations of CH_2_ and CH_3_ groups of phospholipids and proteins.

A number of peaks observed are assigned to amino acids. First of all, the sharp band at 1003 cm^−1^ (±3 cm^−1^) can be assigned to ring breathing mode of Phenylalanine (Phe) of protein. This band is not sensitive to conformational changes of protein and therefore can be used for normalization of the Raman spectra of protein^41,42^. Bands at 1030 cm^−1^ and 1207 cm^−1^ occur due to the C-H in-plane stretch of Phe and the aromatic-carbon stretch of both Phe and tryptophan, respectively. The bands which form the shoulder of the amide I band at 1605 and 1621 cm^−1^ are attributed to Phe and tyrosine correspondingly. Additionally, the bands at 750 cm^−1^, 1130 cm^−1^, 1314 cm^−1^ and 1585 cm^−1^ are well-observed, assigned to a vibrational mode of the pyrrole ring in hemeprotein cytochrome c (Cyt c), which is a small hemoprotein found in mitochondrial membrane and plays a key role in ATP production. Cyt c absorbs light at around 530 nm and therefore shows a strong Raman scattering because of resonant absorption of the 532 nm excitation light.

The dark areas observed in cytoplasm reveals intense lipid Raman signal (shown as spectra Fig. 3a), presumably are a result of the lipid aggregation in the cytoplasm to lipid droplets^43^ (analogous to lipid reach regions previously reported^23^, below we will call these areas lipid-reach areas, LRA). In the Fig. 3b,c, spectra from light cytoplasm and nuclei are presented, respectively. Note the spectra can be compared with spectra from other living cells^6^. The spectra of nucleus of the embryo are comparable to other cells’ nucleus, spectra of the embryo cytoplasm are similar to the cell cytoplasm, and the spectra from LRA in the embryo cytoplasm are similar to spectra from cellular compartment (Golgi apparatus, mitochondria, endoplasmic reticulum) presented in literature^6^. The spectra can be compared also with the spectra of different areas of living non-fixed embryo obtained from 2D Raman mapping and analyzed to distinguish different cellular components using K-means clustering^26^. Principle Component Analysis (PCA) was applied to the spectra to compare groups of spectra for LRA and cytoplasm. The PC-1 and PC-2 calculated for spectra from LRA of normal developing embryos and for cytoplasm give clear clustering between these groups of spectra to characterize smaller variability between spectra in the group and between the groups (See Suppl. Mater. Fig. 1).

### Raman investigation of fertilized and unfertilized oocytes

**Figure 4:**
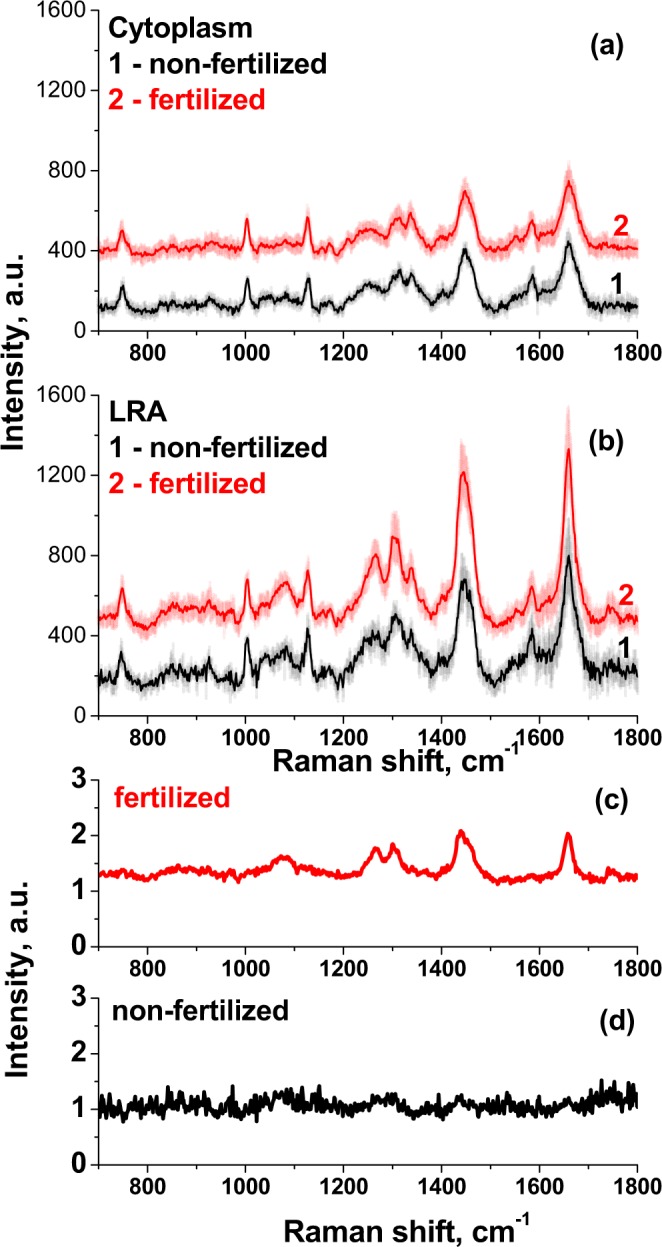
Comparison of the characteristic Raman spectra of non-fertilized (and further non-developing) oocyte (1) and fertilized (further developing) embryo (2), measured from cytoplasm (**a**) and lipid-rich areas (LRA) (**b**). The spectra are normalized to the Phenylalanine (Phe) peak at 1003 cm^−1^. Comparison for fertilized (**c**) and non-fertilized (**d**) oocytes of the ratio (LRA spectrum)/(cytoplasm spectrum). Excitation, 532 nm; power 3 mW; WI objective 60×. The spectra measured for 7 oocytes at the optimal conditions were averaged, separately for every area (cytoplasm, LRA, nuclei).

To evaluate the feasibility of using Raman spectroscopy to analyze the embryo state for prognosis of the embryo ability to develop, the spectra from fertilized zygote and unfertilized oocyte were compared. In Fig. 4, averaged Raman spectra of the unfertilized (and further non-developing) oocyte and fertilized (further developing) one-cell embryo are shown. The spectra from fertilized oocyte don’t differ observably from spectra from developing 2-cell embryo, but differ from spectra of non-fertilized oocyte. The difference between the spectra from LRA (Fig. 4b) of fertilized and unfertilized oocytes is more pronounced than from cytoplasm (Fig. 4a). The spectra shape doesn’t change appreciably, but relative intensities of bands at 1660 cm^−1^ and 1445 cm^−1^ decrease for unfertilized oocytes, as well as the intensities of bands in the 1200–1360 cm^−1^ region, associated with number of lipid bonds vibrations and amide III band. In analyzing the spectra, we found that relation between spectra from cytoplasm (Fig. 4a) and LRA (Fig. 4b) significantly varies for embryos in different state. It is illustrated in Fig. 4c,d where the ratio (spectrum from LRA)/(spectrum from cytoplasm) is presented for fertilized (Fig. 4c) and unfertilized (Fig. 4d) oocyte. Also, to compare spectra from developing embryo and non-fertilized ova, PCA was applied to the group of spectra from LRA of the embryo and oocytes. The PC-1 and PC-2 score plot for 2 spectra sets from LRA of normal developing embryos and from LRA non-fertilized oocyte (see Suppl. Maters_Fig. 2) confirms the difference between the spectra.

### Raman spectroscopy of UV irradiated embryos

**Figure 5:**
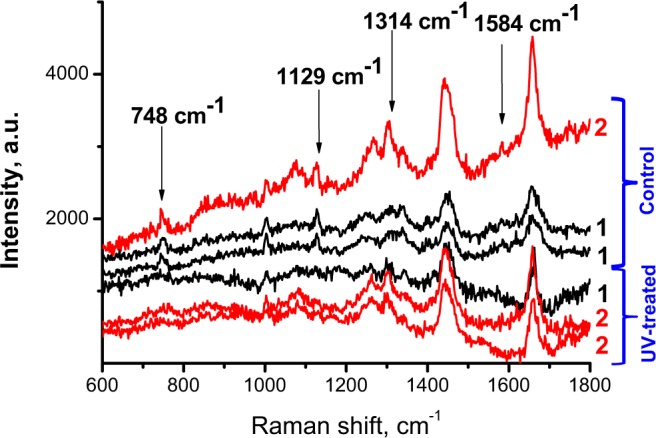
Effect of UV irradiation on Raman spectra of embryo, comparison of spectra of non-treated (control) and UV-treated embryo. (1) Cytoplasm, (2) LRA, arrows indicate Raman spectral peaks of Сyt c. Excitation 532 nm; power 3 mW; WI objective 60x.

To further demonstrate the estimation of the embryo state, the embryos were subjected to UV irradiation (with 325 nm wavelength laser) to stimulate photochemical changes. In Fig. 5, Raman spectra measured from UV-treated 2-cell embryos are shown and compared with the spectra of non-treated embryo. The most pronounced difference is observed in spectra of LRA, in the 1200–1360 cm^−1^ region, associated with number of lipid bonds vibrations and amide III band. This interval also includes vibrations of Phe and tryptophan. Significant decrease in all Cyt c bands is also clearly observed. The changes in spectra of nuclei and cytoplasm were less pronounced, but qualitatively similar.

## Discussion

The aim of this work is to determine the optimal conditions and parameters of Raman spectroscopy applicable for studying live embryos without impacting their development *in vitro* up to blastocyst. The ability to develop *in vitro* till latest preimplantation stage, blastocyst, is the main and most important criterion of success in all types of manipulations with mammalian oocytes and early embryos *in vitro*. Blastocyst is the last stage which mammalian embryos can reach outside the mother’s body and the ability of embryos to develop till blastocyst is the strictest assessment of their quality possible *in vitro*. This ability correlates well with embryo ability to develop *in vivo*^36,37^. Therefore, this is the most important and necessary step of evaluation of embryo quality and developmental ability *in vitro* before embryo transfer to foster mother with the aim to estimate viability and ability to develop to a live offspring *in vivo*. In addition, the Mouse Embryo Assay (MEA), growing mouse embryos from the zygote or 2-cell to the blastocyst stage, is the most widely used bioassay for the estimation of general culture conditions and toxicity in any products manufactured in use by IVF clinics^44^.

Previously, optimization has been attempted by minimizing the measuring spectral range to decrease laser exposition time and influence on EME and oocytes. In general, for laser methods applied for living mammalian oocyte or embryo estimation and investigation, the limiting factor is the effect of the laser irradiation on the embryo. The effect can depend on the irradiation power, wavelength, exposure time as well as the embryo age and state as has been shown in some studies on the laser effects on embryo. Only a few works combining the analysis of the irradiation effect with optical-spectroscopic methods of the embryo investigation have been reported^39,45^. Using low-level laser irradiation still affected granulosa cells in a time-dependent manner that may result in changes in the oocyte but no changes in meiotic progression or embryo production have been observed^46^. One of the most probable mechanisms of light photodamage, including lasers and other kinds of laboratory/microscopic light effects, is the reactive oxygen species (ROS) production^18,45^. Variations on the laser irradiation parameters allow selection of the most suitable conditions for laser manipulation of cells depending on subjects of studies^47^. Shifting the excitation to longer wavelength (up to IR) can be safer for embryos. It is then reasonable to suggest that using optimal parameters of laser irradiation the embryos will preserve their developmental ability after the treatment. Indeed, using multi-photon laser scanning microscopy (LSM), with 800–1047 nm wavelength laser excitation instead of one-photon imaging technique (with excitation laser wavelengths 514–568 nm) has been shown less harmful to living hamster embryos^45,48^. Hamster embryos with fluorescence labeled mitochondria repetitively imaged by two-photon LSM are subsequently capable of blastocyst and even fetal development^48^; whereas confocal imaged embryos don’t reach the morula stage.

Most previous Raman studies have been performed on fixed mammalian oocytes and early embryos^22–24^ and have found detectable biochemical variation within the oocyte during its maturation and development^24^. Particularly, the method to estimate the oocyte maturity from Raman spectra, revealing the ratio of protein to lipid in the oocyte cytoplasm has been suggested^23^, based on Raman mapping distribution of certain chemical species in the oocyte. Aggregation in clusters and asymmetrical localization of macromolecules in the mature oocyte was observed similar to cytoplasmic polarization in the eggs of lower organisms. Raman spectroscopic analysis, including mapping is presented as analytical tool for the oocyte studies, however, the fixation procedure affects the object which is not favorable for studying biochemical processes^26^, and even more important for applications, not to mention estimating embryos for IVF procedure.

In general, the Raman spectra from fixed oocyte samples are comparable to spectra from living mice oocyte^24^. However, difference has been observed from detailed analysis comparing the spectra from different areas and structures of living and fixed embryo^26^; particularly, aldehyde fixation can change the oocytes structure and molecular composition. In the studies of living embryo the variations in the spectral peaks positions and relative intensity in the living embryo spectra can give more complete information about the ova state^27^. Spectra from young oocytes were found distinguishable from those obtained from *in vitro*-aged, oxidative-damaged and old oocytes. Biochemical modifications of cortical F-actin in oocyte^29^ and conformational changes in protein and glycoprotein secondary structure in the zona pellucida^29^ have been observed at cryopreservation of matured ovine oocytes.

However, the most recent Raman studies of mouse live oocytes^26^ and embryos^34^ didn’t include evaluation of ova quality both before and after investigation. Therefore, it is not possible to discuss the method’s safety for the samples as well as to suggest whether the obtained results present real information about ova structure and composition, or they are the consequence of photodamage as a result of the laser measurement. Moreover, study of frozen ova immediately after thawing^34^ can be complicated by the ova incubation for vitrification, which has been done with mixture of cryoprotectants such as DMSO, acetamide and propylene glycol at very high concentrations. It’s well known that particularly DMSO and acetamide can initiate the processes in living ova affecting Raman spectra. For instance, biochemical modifications of cortical F-actin in oocyte^29^ and conformational changes in protein and glycoprotein secondary structure in the zona pellucida^28^ have been observed by Raman at cryopreservation by vitrification of matured ovine oocytes. Additionally, it’s known that embryos (especially at early stages before 8-cell) can perish after cryopreservation. So for Raman analysis of living embryo, the developmental ability of ova after thawing by *in vitro* cultivation should be checked. Therefore, although Raman spectroscopy as tool for embryo investigation has been presented previously^22–25,27–29^, the results couldn’t be considered for analysis of embryos state and prognosis for further development. Correspondingly, no selection of optimal parameters of non-destructive laser treatment has been suggested.

In this study we analyzed the conditions and parameters of laser treatment on Raman spectroscopic measurements of living embryos which allow acquisition of Raman spectra suitable for further analysis and at the same time don’t affect the embryo vitality. Although, in general, we have used less favorable excitation wavelength in visible range (532 nm) than, for example using IR (785 nm)^26^, we have been able to select safe parameters for the living embryo measurements. Using 532 nm wavelength laser the safe conditions of the experiment can be selected; while using 488 nm wavelength excitation (Raman spectrometer α-SNOM, Witec, Germany) even when the laser power was decreased to less than 1 mW, after the measurement, no living and developing embryos were observed (data not shown).

We have found that developmental rate from 2-cell to blastocyst stage did not significantly differ between Raman-treated and control embryos (Table 1). However, Raman investigation of 2-cell embryos can decrease cells number in blastocysts developing from these embryos (Table 2). As the cell number in blastocysts is considered as one of criteria of embryo quality, therefore, our results still demonstrate some possible negative effect of laser investigation on embryo’s further development. The reason of cell number reduction in blastocysts after Raman exposure is still unclear. We can propose that this is the result of proliferation rate decreasing at early stage embryos or apoptosis increasing. However, we can’t exclude that both processes are involved and led to the cell loss. Additional experiments are needed for understanding the mechanisms of laser impact on preimplantation embryos. We believe that further optimization of Raman protocol will allow to avoid this negative effect. On the other hand, cell number reduction does not obviously mean decreasing embryo viability. Different mammalian embryos have demonstrated a high ability for embryonic regulation at preimplantation stages. For example, microsurgical splitting of bovine, goat, porcine, and murine morulae does not adversely affect embryonic development or birth rate^49–52^. Removing 3–7 cells from human morulae at biopsy also does not adversely affect embryo development *in vitro* or *in vivo*^53^. Therefore, further study is needed for estimation of Raman investigated embryos viability *in vivo* after transfer to foster mothers. The knowledge of safe conditions at the Raman measurements is necessary both for applications and for embryological research, including studies of developmental mechanisms, for understanding the state of the studied ova during measurements, to distinguish the processes at development and maturation from destroying/degradation under laser. Thus, any detailed analysis of the Raman spectra in accordance with the embryo morphology, discussed in terms of the embryo functionality and development should be accompanied with an analysis of the laser treatment safety.

As we aim to suggest and analyze the applicability of Raman-based method for embryo state evaluation, we need to develop the characteristic estimation criteria and simultaneously to decrease exposure of the laser treatment and simplify the estimation procedure. Analysis of Raman spectra obtained on different areas of live 2-cells mice embryos and oocytes shows the characteristic bands for most living cells^38,39^, as well as the comparable spatial distribution of their intensity along the embryo^27^. The spectra of nucleus of the embryo are comparable with the human glioma cell nucleus. Low intensity spectra of the embryo cytoplasm are similar to the cell cytoplasm; the high intensity spectra from lipid-rich structures (LRA) in the embryo cytoplasm are similar with spectra from cellular compartment (Golgi apparatus, mitochondria, endoplasmic reticulum)^6^. Variations in the intensities of some of these bands at various embryo states are observed and can be used to develop criteria for the embryo quality and viability estimation (Fig. 4c,d). The ratio of (spectrum I)/(spectrum II) for fertilized embryo, which continue to develop, shows clear difference between these two spectra in both, lipids and proteins (Amide I, III) characteristic ranges, while for unfertilized ova the ratio shows, in the whole range, some variations comparable with noise level, and is difficult to identify. One can suggest that changes in this ratio correlates with the structural and chemical changes which are connected with the embryo loss of viability and can be used to develop method and quantitative criterion for the estimation of the embryo state and prognosis of its further development. Note that lipid metabolism of ova now attracts much attention of researchers, for analysis of early development^54–57^. Therefore, the difference between spectra of individual embryos inside of every group is lower than differences between average spectra of groups of developed, fertilized embryos and non-fertilized ova.

Significant decrease in Cyt c Raman bands is observed after the embryo irradiated with UV light (Fig. 5); The cellular damaging of UV irradiation, first of all is the DNA damage (including mitochondrial DNA) and mutagenesis, are in high degree mediated by reactive oxygen species (ROS)^18,19^. Significant role of mitochondria in the cellular response to UV has been frequently discussed up to recent studies^58^. Particularly, it has been shown that the oxidative stress in mitochondria membrane entails cytochrome translocation into cytozole^48^. Thus, we can suggest that decreasing Cyt c Raman bands after the embryo UV irradiation can be related to oxidative stress mitochondria dysfunction^59^. The Cyt c release can be result of different pathological states of cell, including initiation of programmed cell death, apoptosis; released Cyt c acts as a trigger of caspase cascade activation, resulting in the initiation of apoptosis before morphological changes in the apoptotic cell are visible, but the cell gradually perishes. On the other hand, it has been shown that decreasing Cyt c peaks can be result of the cytochrome release into cytoplasm and changes in Cyt c redox status, from reduced to oxidized form^58,60^, in which also the formation ROS under UV treatment can contribute.

The observed spectra also allow analysis in the amides spectral ranges, for example, in the 1230–1330 cm^−1^ range, associated with amide III band. The amide III band is the in-phase combination of NH in-plane-bend and CN stretching, with small contributions from CC stretching and CO in-plane-bend. Like amide I band, amide III is conformationally informative and exhibits a distribution of bands consistent with a mixture of secondary structures, including α-helix, β-sheet and random coil. The analysis can be facilitated by very complex character of the amide III, however the observed spectral transformations in this range are significant and also can be considered for viability criteria development^40,61^, as the conformational changes are closely connected with the protein functionality and correspondingly, with correct development of the embryo.

One can see that different disorders in the embryo caused by different reasons (non-developed of non-fertilized oocyte or degraded UV-treated oocytes in our case) can be observed due to their different influence on the spectra. It allows analysis of the corresponding effects and treatments in terms of the embryo molecular composition and it is important for development of certain criteria of the oocytes and embryo estimation, including the criteria which could be practically used in IVF techniques.

Although in this work, safe conditions of the irradiation using laser with excitation wavelength 532 nm have been selected, one can suggest that these conditions still can be improved. As these conditions should allow Raman analysis of the EME with its further normal development, further steps on optimization of safe Raman analysis of live embryo state can include (1) using longer wave excitations (safer for EME), (2) optimization and decreasing of tested areas in the embryo; (3) the selection of the discrete spectral ranges with the most informative Raman bands; (4) searching ways to decrease the exposition time without the information loss.

## Conclusion

The presented results demonstrate for the first time the possibility of Raman measurements on live EME while maintaining the embryo’s viability. The laser power and exposure were varied and the embryo viability after the measurement was estimated via observation of further development to select the most achievable optimal conditions of the measurements. Based on the embryo evaluation, the safe conditions for Raman investigation have been selected. For the 532 nm wavelength laser excitation, the safe laser power was up to 3 mW, exposure time 10 sec and the spectra acquisition times 3–4 resulting in total irradiation time 40–55 sec; the number of the spectra measured from one embryo were 4–6 (or, for one blastomere 2–3 spectra). The obtained Raman spectra are sufficient for detection and analysis of main cell chemical components and allow us to suggest criteria for estimation of the embryo state and prognosis of its development. Additionally, we have shown that the methods of 2-photon fluorescence microscopy can be adjusted and used for the development estimation together with conventional microscopic morphological evaluation. At the same time, it is still quite complicate to distinctly interpret the EME Raman signals and their connection with the embryo state. Additional investigations on embryos of various stages and states are needed for understanding how Raman spectra correlate with the dynamic of biochemical processes in developing embryos and how these spectra can be used for estimation of the embryo viability and developmental potential. With this aim it should be worthwhile to perform some Raman measurements on the same developing embryo or/and on embryos with synchronized development. We consider the described method of Raman spectroscopy will be helpful for investigation of fundamental mechanisms of mammalian’s early development and for application in medical embryology.

## Materials and Method

### Ethics Statement

All experiments were performed according to the guidelines for the human use of laboratory animals by the Laboratory Animal Center of the National Dong Hwa University (NDHU), Taiwan, and all procedures were approved by Institutional Animal Care and Use Committee (IACUC) of Laboratory Animal Center of the National Dong Hwa University, protocols # 001.

### Animals

The study was carried out on mice (CBA/Ca and C57BL/6) provided by National Applied Research Laboratories of National Laboratory Animal Center of Taiwan, (Taipei, Taiwan). Total 7 CBA males were used for mating and 21 C57Bl females were used for embryos and oocytes obtaining. At the time of experiment, the mice were 6–10 weeks old. C57BL/6 female mice were superovulated by the standard method of intraperitoneal (i. p.) injection of 7 IU pregnant mare’s serum gonadotropin (PMSG) (Intergonan, MSD, Germany) followed by an i. p. injection of 7 IU human chorionic gonadotropin (hCG) (Ovogest, MSD, Germany) 48 hours later. After hCG injection, females of one group were used for obtaining oocytes, females of the other group were placed in cages with CBA/Ca males and examined the following morning for the presence of a vaginal plug (day 1 of pregnancy). Mice were fed with standard laboratory diet, provided water ad libitum.

### Embryos recovery and culture

The zygotes and oocytes were recovered from the excised oviducts (15–18 hr after the hCG injection) by dissection of oviduct wall into M2 medium (Sigma, USA) containing 0.1% (w/v) hyaluronidase (Sigma, USA) to remove cumulus cells. *In vivo* produced 2-cell embryos were recovered from the excised oviducts (45–48 hours after hCG) by M2 medium flushing via oviduct ampulla using fine capillary connected with syringe. Then the ova were washed in M2 medium and used for manipulations. For *in vitro* cultivation, 2-cell embryos were transferred to four-well culture dishes (Nunc, Denmark) and cultured in M16 medium (Sigma, USA) under 5% CO_2_ in air at 37 °C. Previously, the culture medium was equilibrated with the gas phase and temperature in a CO_2_ incubator for 2–3 hours. The same method of cultivation was used for estimation of embryo viability after laser treatment.

### Manipulation of ova for Optical-Spectroscopic studies

For Raman spectroscopy, oocytes and embryos were thoroughly washed with modified Dulbecco’s phosphate buffered saline (PBS, Sigma) without phenol red and macromolecules. Note that during the spectroscopic measurements both the sample and control embryos were kept in the equal conditions in PBS, at room temperature in ambient atmosphere for time necessary for the measurements, which was total in the range of 10–30 min). Total 77 embryos from different mice were used for Raman measurements at various conditions, 79 embryos for development control, 6 embryos for UV-treatment, and 14 non-fertilized oocytes.

### Raman measurements

For Raman spectral measurements special chamber was made with silicon (Si) wafer as substrate, where the embryos were placed in the 0.5 ml drop of the same PBS medium. The Raman signal was collected in the reflection mode, so the Si wafer substrate is the most convenient sample support. The Si is used for minimizing the loses at collection of Raman signal and for minimizing background signal, because Si has the Raman spectrum with a sharp peak at 520.5 cm^−1^ and which can be easily distinguished from the spectrum of the measured sample. On the Si substrate a dimple was etched to prevent the embryo floating in the medium during the measurements.

A micro Raman spectrometer (Renishaw, UK) with a grating (1800 1/mm) coupled with a microscope (Leica, Germany) and a 532 nm wavelength laser was used as the excitation for the spectral acquisition. Water-immersion objective LUMPlanFl 60×/0.9 (Olympus, Japan) was used for measurements.

For the selection of the safe conditions of the measurements, the power in focal spot was varied in the range of 2.8–6.0 mW. The light was focused on the selected area of the sample (inside blastomere). Due to light scattering by the blastomere’s cytoplasm/organelles the irradiated area size was larger than minimal size of focal spot, which is estimated ∼1 μm diameter for the objective used. To search the safety conditions of the measurements the acquisition time and number of scans of spectra measured from one embryo were changed between 10 sec × 2 to 10 sec × 10. We found the laser power of 3 mW, exposure time 10 sec, the spectra acquisition times 3–4 resulting in total irradiation time 40–55 sec, and number of the spectra measured from one embryo were 4–6 (or, for one blastomere 2–3 spectra) appear to be the safe conditions for this investigation. The same number of spectra was measured from every blastomere of the studied embryo. Total 27 embryos were examined at these conditions. Additionally, 14 non-fertilized oocytes were investigated at the same conditions.

To reveal the potential of Raman spectroscopic investigation to estimate the embryo state the embryo after harmful treatment and non-fertilized oocytes were investigated at the established safe conditions. For that 6 embryos were treated with UV irradiation. The Petri dish with drop of medium with embryo has been positioned on the microscope stage and the irradiation He-Cd laser (Kimmon Koha, Japan) with 325 nm wavelength at 5 mW/cm^2^ average power density and 120 sec exposure has been directed to the embryo. Their Raman spectra were measured directly after UV-treatment. The background spectra due to medium and substrate were measured for every sample. The medium and substrate weakly affected the embryo spectra as the laser was focused inside a sample, relatively large object such as the embryo (100–120 μm). The background correction for the medium and substrate was performed when needed. Additionally, some embryos could exhibit their own fluorescence signal due to laser irradiation. Baseline correction was performed to subtract the fluorescence signal, which could depend on metabolic state and also can be used for the embryo analysis and evaluation, but in the present work it is hampering the analysis of Raman spectra. For that Renishaw spectrometer software and Origin software were used. The spectra measured for the same morphological areas were considered individually or averaged for the analysis peculiarities and identification of common patterns. Principle Components Analysis (PCA) was applied to the spectra after background/baseline correction and normalizing to peak 1003 cm^−1^ (Phe) to compare sets of spectra for different samples. For the PCA Unscrambler *X*10.5 software was used.

### Embryo evaluation

The developmental rate of embryos after the Raman measurements are compared with control embryos developed at the same conditions without any laser irradiation. The development was characterized via estimation of the embryo ability to reach morphologically normal blastocyst stage and counting the cell number in the embryo. To count the cell number, the blastocysts developed *in vitro* during 72 h were stained by 10 min incubation in M2 medium with 10 µg/ml specific DNA fluorescent dye Hoechst 33342. For imaging of stained nuclei and quantitative count of cells (in blastocyst stage), two photon excitation using a 740 nm wavelength Ti-sapphire femtosecond laser (Chameleon Ultra II, Coherent, USA) was used. Scanned image was registered using an Olympus IX71 microscope (with a Semrock 447/60 filter). A two-dimensional scanner (EINST Technology, Singapore) was used for imaging. The registration was done with single photon counting system (PicoHarp 300, PicoQuant Germany) and cooled photomultiplier (PMA-C 192-N-M, PicoQuant, Germany).

Developmental rates and significant difference in the number of cells for the Raman measured and control embryos were analyzed statistically by Student’s t-test and Z-test. A value of P < 0.05 was chosen as an indication of statistical significance.

## Supplementary information

Supplementary Materials
