# Supplementary material for: Raman spectroscopy on live mouse early embryo while it continues to develop into blastocyst in vitro
Source: Sci Rep. 2019 Apr 29;9:6636. doi: 10.1038/s41598-019-42958-5 (PMC6488652; doi:10.1038/s41598-019-42958-5)
Supplement: Supplementary file 1 — Supplementary Materials [file 41598_2019_42958_MOESM1_ESM.pdf]

Supplementary Materials
SREP-18-19063B

Raman spectroscopy on live mouse early embryo while it continues to develop into blastocyst in vitro

Elena Perevedentseva[1, 4], Alexander S. Krivokharchenko[2], Artashes V. Karmenyan[1]*, Hsin-Hou Chang[3]*, Chia-Liang Cheng[1]*

[1] Department of Physics, National Dong Hwa University, Hualien, Taiwan
[2] N. N. Semenov Institute of the Chemical Physics, Russian Academy of Sciences, Moscow, Russia
[3] Department of Molecular Biology and Human Genetics, Tzu Chi University, Hualien, Taiwan
[4] P. N. Lebedev Physical Institute of the Russian Academy of Sciences, Moscow, Russia

Correspondence and requests should be addresses to CLC (email: clcheng@gms.ndhu.edu.tw) or AVK (email: artashes@gms.ndhu.edu.tw)

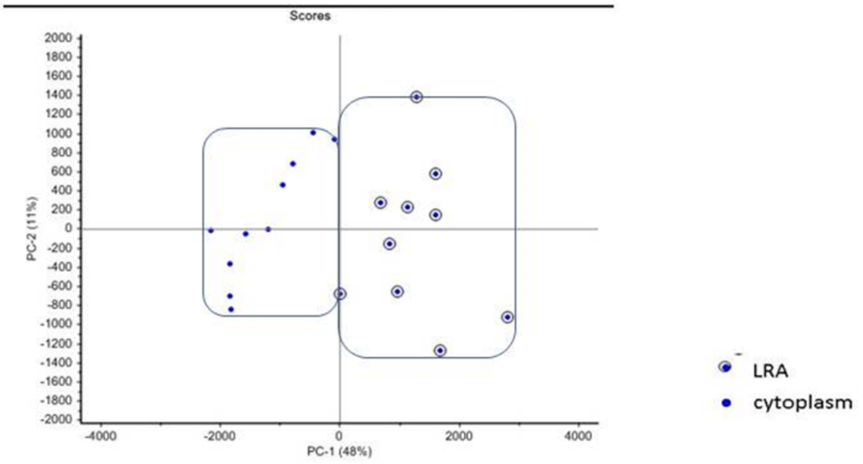

Fig. 1 Score plot of PCA performed for datasets for LRA and cytoplasm of developing embryo.

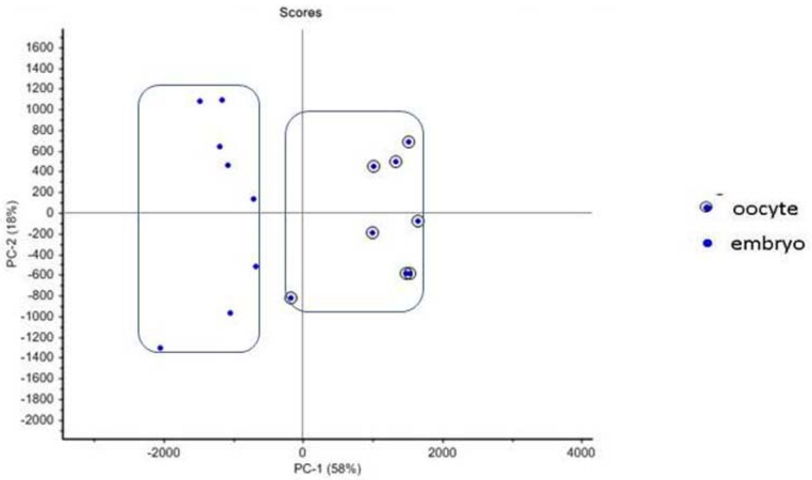

Fig. 2 Score plot of PCA performed for datasets for LRA of developing embryo and oocyte.
